# Advances in understanding the immunity of the brain and its borders: Focus on brain macrophages

**DOI:** 10.1002/ctm2.70014

**Published:** 2024-08-27

**Authors:** Patrick Süß, Martin Diebold, Roman Sankowski

**Affiliations:** ^1^ Department of Molecular Neurology University Hospital Erlangen, FAU Erlangen‐Nürnberg Erlangen Germany; ^2^ Institute of Neuropathology, Faculty of Medicine University of Freiburg Freiburg Germany

**Keywords:** brain border regions, development, glioblastoma, immunity, macrophage, turnover

## Abstract

A recent study outlines the phenotypes of brain border region macrophages in developing, normal and glioblastoma‐affected brains. For the first time, the authors show in‐vivo turnover of human brain border macrophages. The findings have implications for the understanding of brain border immunity and potential macrophage targeting therapies.

**keypoints:**

- Human border region macrophages are distinct from microglia.
- These distinct phenotypes are established early during embryonal development ‐ Brain border macrophages are partially replaced by bone marrow‐derived myeloid cells.
- The transcriptional phenotypes of glioblastoma‐associated macrophage are determined by the anatomical region.

1

The perivascular space, the meninges, and the choroid plexus (CP) are highly specialized niches strategically located between the central nervous system (CNS) and the periphery of the body. They are pivotal interaction sites between the immune system and the brain, serving as gateways for blood‐derived immune cells to enter the CNS under pathological conditions. These niches harbour rare resident immune cell populations known as CNS‐ or border‐associated macrophages (CAMs or BAMs). A recent study provides a comprehensive profiling of CAMs and other immune cells at the CNS borders in normal brain, development, and glioblastoma.^1^

CAMs have diverse immunological and tissue maintenance functions. Being directly exposed to peripheral stimuli, they respond to inflammatory and chemical cues like bacterial metabolites,^2^ and mechanical influences like arterial hypertension.^3^ These cells are ideally poised to mediate systemic immune effects on the CNS. Under normal conditions, perivascular macrophages regulate cerebral blood flow.^4^ Moreover, recent animal studies implicate CAMs in various neurological diseases. In experimental autoimmune encephalomyelitis (EAE), a mouse model for multiple sclerosis, CAMs adopt reactive states.^5^ In a Parkinson's disease model, CAMs facilitate T cell recruitment and activation.^6^ In an Alzheimer's disease model, CAMs were implicated in Amyloid‐β clearance.^2^ These studies highlight the initial understanding of the diverse functions of CAMs. As the most abundant immune cell of the CNS borders, many more functions remain to be explored.

Analyses of human CAMs are hampered by their small population sizes and limited accessibility due to their complex integration into the extracellular matrix. Novel single‐cell technologies were instrumental in understanding CAM phenotypes. Given their intricate interaction with their surroundings, the combination with spatially‐resolved transcriptomic technologies is crucial for a more comprehensive understanding of these cells.^7^ While the previous knowledge about CAMs was based on rodent studies, the development, transcriptional signature, turnover, and function of human CAMs during homeostasis and disease remained largely unexplored.

Our recent study, utilizing cutting‐edge single‐cell multi‐omics and spatial transcriptomic analyses, provides the first in‐depth characterization of human CAMs.^1^ We found an evolutionary conservation between human CAM subsets and their previously profiled mouse counterparts.^8^ These subsets include macrophages in the dura mater, leptomeningeal macrophages, perivascular macrophages, macrophages in the CP stroma, and Kolmer epiplexus cells (Figure 1). Our findings reveal common and site‐specific CAM signatures, highlighting their complex spatial organization and high degree of specialization.

**FIGURE 1:**
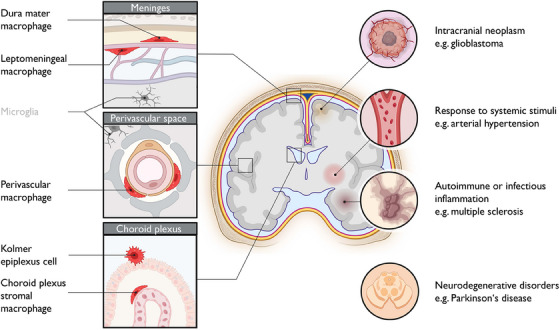
Central nervous system (CNS)‐associated macrophages, their locations and roles in pathophysiology. Schematic representation of subsets of CNS‐associated macrophage (CAMs) (red) and microglia (grey), showing their location within the CNS border regions (left) as well as their contribution to various diseases (right). The figure was generated using BioRender.com. It was adjusted from: https://idw‐online.de/de/news826339.

We analyzed macrophage turnover across various anatomical niches using post‐mortem tissues from female patients who underwent peripheral blood stem cell transplantation (PBSCT) from male donors. This approach allowed us to track Y‐chromosome‐positive macrophages as a metric of turnover. Previous mouse studies have identified microglia, perivascular and leptomeningeal macrophages as well as Kolmer epiplexus cells as long‐lived and self‐renewing subsets, while dural and CP stromal macrophages exhibited lifelong turnover by blood‐derived myeloid cells.^8^, ^9^ Our study demonstrates site‐specific turnover dynamics of human CAMs through the engraftment of myeloid‐derived cells labelled with Y‐chromosome in‐situ hybridization. We observed considerable turnover across microglia and CAM niches following PBSCT, with compartment‐specific differences consistent with previous mouse data. Choroid plexus and dural macrophages exhibited shorter half‐lives (T50 = 62 days and T50 = 120 days, respectively) while microglia had the slowest turnover (T50 = 265 days), followed by leptomeningeal (T50 = 198 days) and perivascular macrophages (T50 = 150 days). Here, the T50 indicates the average duration until 50% of the resident macrophages have been replaced. These findings highlight region‐dependent macrophage turnover dynamics across different anatomical compartments.

The human brain develops well into the fourth decade of human life, during which microglia and CAMs play significant roles. We investigated the developmental profiles of microglia and CAMs using single‐nucleus RNA‐sequencing of fetal CAMs at different developmental stages. Our findings revealed a distinct CAM signature from microglia even during embryonic development, characterized by enhanced expression of genes such as LYVE1, CD163 and F13A1.^1^ While microglia displayed marked developmental dynamics consistent with brain tissue growth and refinement, CAMs appeared relatively stable. Gene ontology analysis revealed that developmental CAM phenotypes were strongly influenced by hypoxia, as fetal oxygen supply is solely from maternal circulation. Thus, CAMs exhibit a gene expression signature that is distinct from microglia and stable throughout development.

In glioblastoma, the most frequent malignancy of the CNS, the cellular immune microenvironment consists of a diverse array of resident and infiltrating myeloid and lymphoid cells. Previous studies, including our own, have shown that these immune cell subsets interact with each other and with brain cells to create an immunosuppressive microenvironment.^10^ However, the specific contribution of CAMs in this complex immune landscape was not well understood. We conducted anatomical dissections of glioblastoma‐associated leptomeninges and tumor cores. Through the integration of single‐cell gene and surface protein expression, spatial transcriptomics, and mass cytometry, we revealed a comprehensive glioblastoma‐associated myeloid cell landscape. Spatially resolved transcriptomic analysis unveiled differential composition in the peritumoral region, hypoxic, and necrotic tumour areas. Notably, immune cells in the leptomeninges exhibited gene expression patterns associated with cell migration, consistent with the role of the leptomeninges as a gateway into the tumour. Overall, our study elucidates the finely orchestrated dynamics of glioblastoma‐associated immune cells, which are influenced by the hypoxic tumour environment.

In summary, our study has significantly enhanced the understanding of CAM diversity, turnover, and function in humans through the integration of several highly sensitive technologies. These new insights into human CAMs offer considerable potential for future clinical applications. The identified human CAM signatures will facilitate specific approaches for analyzing and targeting these cells. Additionally, our findings on the turnover dynamics of human CAMs and the demonstration of blood‐derived myeloid cell integration into CAM niches provide a foundation for future cellular replacement approaches in the growing spectrum of CAM‐related diseases.

## AUTHOR CONTRIBUTIONS

Roman Sankowski conceptualized the research highlight. Patrick Süß, Martin Diebold and Roman Sankowski contributed to writing and preparing the figure. All authors have read and approved the article.

## CONFLICT OF INTEREST STATEMENT

The authors declare no conflict of interest.

## ETHICS STATEMENT

None.
